# Germination Responses of Ryegrass (Annual vs. Perennial) Seed to the Interactive Effects of Temperature and Salt-Alkali Stress

**DOI:** 10.3389/fpls.2018.01458

**Published:** 2018-10-09

**Authors:** Jixiang Lin, Xiaoyu Hua, Xiaoyuan Peng, Bolin Dong, Xiufeng Yan

**Affiliations:** ^1^Alkali Soil Natural Environmental Science Center, Northeast Forestry University/Key Laboratory of Saline-Alkali Vegetation Ecology Restoration, Ministry of Education, Harbin, China; ^2^Department of Plant Pathology, North Carolina State University, Raleigh, NC, United States

**Keywords:** annual ryegrass, perennial ryegrass, temperature, salinity–alkalinity, germination recovery

## Abstract

Ryegrass is considered a useful grass species for forage production and turf purposes. Annual ryegrass (*Lolium multiflorum* Lam.) and perennial ryegrass (*Lolium perenne* L.)are two species of ryegrass with similar genomes. So far, little information exists concerning their physiological response to salt-alkali stress during germination stage, especially under different temperature regimes. Seeds of ryegrass were germinated at four alternating temperatures (10–20, 15–25, 20–30, and 25–35°C) with salinity (NaCl) and alkalinity (Na_2_CO_3_, high pH) stresses. Results showed that optimal germination for both species under stress conditions occurred at higher temperatures (20–30°C for annual ryegrass; 20–30°C and 25–35°C for perennial ryegrass). Germination percentage and germination rate were both inhibited by increasing salinity or alkalinity, particularly higher alkalinities under any temperature. The inhibitory effects of the high salinity on germination were greater at 10–20°C for both species. However, seeds were subjected to more stress at 25–35°C under alkali stress even though the concentration was very low. In addition, both high and low temperatures lead to a markedly decrease in seed germination under alkali stress for perennial ryegrass. Recovery percentage of both species were highest at 400 mM salinity and 25 mM alkalinity under any temperature, especially 10–20°C, and 25–35°C also resulted in lower recovery percentages under both stresses for ryegrass. Moreover, annual ryegrass had a much higher recovery percentage than perennial ryegrass under such stress conditions. These results suggest that salinity stress and alkalinity stress are greatly different, and the salt-alkaline tolerance of ryegrass seeds is greatly affected by the interactions of temperature and salinity–alkalinity.

## Introduction

Soil salinization and alkalization have been considered as major environmental threats to the entire terrestrial ecosystem (e.g., forest, grassland, agricultural and urban), which not only inhibit plant growth but also lead to a further soil degradation (Lin J. et al., 2014; Guo et al., 2015). In China, approximately 7 million hectares (ha) of land area are affected by salinity. Additionally, in the arid and semi-arid areas of northeastern China, alkalinized land has exceeded 70% of the total area and is still expanding (Wang et al., 2011).

Seed germination is the initial and one of the most pivotal stages in plant life cycle (Bhatt and Santo, 2016). In natural habitats, seed germination and the following seedling establishment phases are always affected by many environmental factors such as soil salinity–alkalinity, light, temperature fluctuations and water availability (Keblawy et al., 2017). Temperature and soil salinity–alkalinity are considered to be the main limiting factors during germination stage for most plant species in the Northeast of China (Lin and Tang, 2005).

Previous studies have clearly shown that seed germination percentage was always very high under non-salinity condition but decreased with the increasing salt concentration in the soil (Khan and Gulzar, 2003; Qu et al., 2008). Salt reportedly inhibited seed germination through osmotic effects (creating a low osmotic potential), ion toxicity (Na^+^, Cl^-^, etc.) or combination of the two effects (Munns, 2002; Zhang et al., 2012). Soil salinity, on the one hand, can delay the germination process. On the other hand, if the salt concentration in the soil is beyond the tolerance limit of plant species, germination behavior would be completely suppressed (Ungar, 1991; Al-Hawija et al., 2012).

Alkalinity is also an inhibiting factor for seed germination in the Northeast of China (Guo et al., 2009; Zhang and Mu, 2009). Alkaline stress not only has the same stress factors as salt stress, but also adds the high pH impacts, leading to much more serious inhibition, which is greatly differed from salt stress (Yang et al., 2007, 2008). However, to the best of our knowledge, the majority of the reports only emphasized the physiological effects of salt stress, with little attention to the alkaline stress, especially on the seed germination stage (Lin et al., 2011; Lin J.X. et al., 2014).

In addition, soil salinity and alkalinity can interact with temperature fluctuations, thereby modifying the seed sensitivity to the stress conditions (Sugahara and Takaki, 2004; Bhatt and Santo, 2016). Temperature is also a determining factor for seed germination of most plants (Probert, 1992). Understanding the temperature effects on seed germination may be useful to evaluate the germination characteristics or the establishment potential among plant species (Tlig et al., 2008). Non-optimal temperature can affect a series of cytosolic enzymes activity and membrane permeability, which will change the seed germinability (Bewley and Black, 1994). Furthermore, salinity-temperature interactions also have significant eco-physiological implications in terms of seed germination under field conditions (Ungar, 1995).

Earlier studies have indicated that although high salinity reduced seed germination percentage and rate, the detrimental effect of salinity was generally less severe at the optimum temperature (Gorai and Neffati, 2007). For example, the toxic effect of salt stress on the seed germination was found much severe under high temperatures for some plant species including *Atriplex cordobensis* (Aiazzi et al., 2002) and *Leymus chinensis* (Lin et al., 2011); but under lower temperatures for other plants, such as *Aeluropus lagopoides* (Gulzar and Khan, 2001) and *Halopyrum mucronatum* (Khan and Ungar, 2001). Moreover, for some plants such as *Urochondra setulosa* and *Sporobolus ioclados*, the detrimental effect of salinity was severe at both higher and lower temperatures (Gulzar et al., 2001; Khan and Gulzar, 2003). However, little is known about the interactive effects of temperature and alkalinity (high pH) on the seed germination.

In general, salt-alkali concentration in the soil changes in the arid and semi-arid regions of northeastern China due to the irregular rainfall and evaporation. Most seeds have the capacity to recover from the salinity–alkalinity shock and start germination once the salinity–alkalinity decreases, which could happen following the high precipitation (Gul et al., 2013). This may be an adaptive strategy of seed germination to withstand salt-alkali stress. The seeds must remain viable under high salinity stress (Ungar, 1991; Lin et al., 2011). Germination recovery test is often used to assess the ability of seeds that are subjected to high salinity to germinate again when transferred to fresh water.

Annual ryegrass (*Lolium multiflorum* Lam.) and perennial ryegrass (*Lolium perenne* L.) are two different species ryegrass but with similar genomes, and can fully interfertile (Warnke et al., 2004). They are widely distributed in North Africa, Europe, and temperate Asia. Annual ryegrass is primarily used in forage production, and is always grown together with other grass species to improve the pasture quality for feeding (Han et al., 2013; Pan et al., 2017). Although perennial ryegrass is also valued for the high yield and nutritive potentials as forage grasses, it is mainly used for turf purposes, such as golf course, fairways, athletic fields, and home lawns, especially in North America and European areas (Li et al., 2006; Xiong et al., 2007). In addition, both species are salt tolerant and have potential in improving degraded soil. Previous studies have reported seed germination responses of this species to temperatures (Shen et al., 2008), cadmium stress (Fang et al., 2017) with either annual or perennial ryegrass. However, no information is available on germination responses of annual and perennial ryegrass seeds to the interactive effects of temperature and salt-alkali stress. We hypothesize that although annual ryegrass and perennial ryegrass are similar in many respects, even genomes, the two species have different germination requirements, especially when they grow in salt-alkali environment at different temperature regimes.

To test this hypothesis, we investigate (1) whether the seeds of annual and perennial ryegrass show any difference in their germination behavior to temperatures under non-salinity/alkalinity conditions, (2) whether the two ryegrass species show differences in their salt-alkaline tolerance, especially under different temperatures, (3) whether the non-germinated seeds of annual and perennial ryegrass can maintain the viability when transferred from high salinity/alkalinity to distilled water at different temperatures.

## Materials and Methods

### Seed Collection and Storage

Annual and perennial ryegrass seeds were collected from the Grassland Ecosystem Field Station, Northeast Forestry University, Heilongjiang province, China (125°22′E, 46°27′N) in 2017. Seeds were then dry-stored in cloth bags at room temperature for further use (experiments were carried out in October 2017).

### Temperatures and Salt-Alkali Stress Treatments

In order to assess the interactive effects of temperature and salt-alkali stress on seed germination process of ryegrass, four alternating temperatures (10–20, 15–25, 20–30, and 25–35°C) were used (Lin et al., 2011), with a 12-h photoperiod (higher temperatures; Sylvania cool white fluorescent lamps, 200 μmol m^-2^ s^-1^, 400–700 nm) and a 12-h dark period (lower temperatures) in the growth chambers (HPG-400, Harbin, China).

The neutral salt NaCl and alkaline salt Na_2_CO_3_ were applied to the salt and the alkali stress groups, respectively. Four salt concentrations: 50, 100, 200, and 400 mM, and four alkali concentrations: 25, 50, 100, and 200 mM were used in this test. Salt stress groups were labeled with S_1_–S_4_ and alkali stress groups with A_1_–A_4_. The pH values ranged from 6.25 to 6.50 in the salt stress groups and from 11.50 to 11.70 in the alkali stress groups. Although the pH values varied greatly at the same concentration between salt stress and alkali stress, the Na^+^ concentrations were consistent.

### Germination Test

Seeds were firstly surface sterilized in 0.1% mercury chloride for 5 min, subsequently washed with distilled water before used in the experiments. Seeds were germinated on two folds filter paper (12.5-cm) placed in 11-cm-diameter petri dishes with 10 mL of test solution. Three replicates of 50 seeds were used for each treatment and distilled water was used as control. The water level was adjusted daily to avoid changes in salt-alkali concentration due to evaporation (Zhang et al., 2012). Seeds were considered germinated with the emergence of radicle. Seed germination percentage was recorded every day for 8 days. Non-germinated seeds from salinity–alkalinity stress were then transferred to distilled water to assess the recovery of germination, which was also recorded every day for 8 days.

Germination rate was estimated by using a modified Timson index of the germination velocity, Σ*G*/*t*, where *G* is the percentage of seed germination and *t* is the germination time (Lin et al., 2011). The maximum value possible using this index with our data was 100 (i.e., 800/8). The lower values represented a less rapid rate of germination.

The seed recovery percentage was calculated by dividing the number of germinated seeds after being transferred to the distilled water by the number of non-germinated seeds under salt-alkaline stresses (Tlig et al., 2008).

### Data Analysis

Germination data were arcsine transformed before analysis of variance (ANOVA). Data were analyzed using SPSS 13.0. A three-way ANOVA was used to test the effects of ryegrass species, temperature, salinity (alkalinity) and their interactions on seed germination indexes. Tukey’s test was used to assess the significant differences of the germination percentage and rate among temperatures in each salinity–alkalinity treatment of each ryegrass species, and the seed germination recovery percentage among salinity–alkalinity treatments and different temperatures (*P* < 0.05). Regression analysis was also used to clarify the relationship between germination rate and salinity/alkalinity concentration under each temperature of the two ryegrass species.

## Results

### Interactive Effects of Temperature and Salinity on Seed Germination of Ryegrass

**FIGURE 1:**
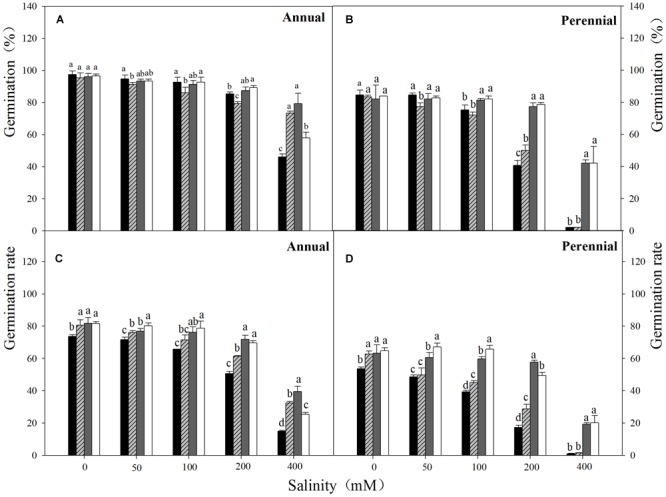
Final germination percentage **(A,B)** and germination rate **(C,D)** of Annual ryegrass **(A,C)** and perennial ryegrass **(B,D)** seeds in salinity stress at temperature regimes of 10–20 (■), 15–25 (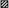), 20–30 (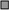), 25–35°C (□). Salt stress: NaCl. Bars represent mean ± SE (n = 3). Different letters indicate significant difference between treatments (Tukey’s test *P* < 0.05).

Three-way ANOVA results showed that seed germination percentage of ryegrass was affected by ryegrass species, temperature, salinity and their interactions (*P* < 0.001, **Table 1**). The highest germination percentage of both species was obtained in non-stress condition (distilled water), and no significant difference was observed between different temperatures. Seed germination percentage decreased with the increasing salinity at all temperatures (**Figures 1A,B**). For annual ryegrass, the optimal temperatures for seed germination were 20–30 and 25–35°C when salt concentration was below 200 mM. However, when salinity reached 400 mM, the highest germination percentage (79.3%) was only found at the temperature of 20–30°C. The inhibitory effect was greater at 10–20°C under the highest salinity, which germination percentage was only 46% in 400 mM salinity. Interestingly, when salinity was below 400 mM, the final percentage was higher at 10–20°C (**Figure 1A**). For perennial ryegrass, the optimal temperatures for germination were 20–30°C and 25–35°C. Unlike annual ryegrass, germination percentages sharply decreased at lower temperatures (10–20 and 15–25°C) when salinity was only 200 mM. At the highest salinity, germination percentages were only 1.8% and 2.3% at these two temperatures (**Figure 1B**). In addition, germination percentage was lower in perennial ryegrass than that in annual ryegrass under treatments.

**Table 1:** Three-way ANOVA for the effects of ryegrass species (SP), temperature (T), salinity (S) and their interactions on seed germination of ryegrass.

	Germination percentage			Germination rate			Germination recovery		
Independent variable	df	Mean-square	*F*-ratio	df	Mean-square	*F*-ratio	df	Mean-square	*F*-ratio
SP	1	12772.0	1268.7***	1	12281.6	2305.9***	1	654.887	10.5**
S	4	8878.4	881.9***	4	10250.8	1924.6***	4	6490.105	104.2***
T	3	990.3	98.3***	3	2008.9	377.2***	3	3605.403	57.8***
SP × S	4	1013.1	100.6***	4	50.9	9.5***	4	165.123	2.6*
SP × T	3	428.3	42.5***	3	265.8	49.9***	3	334.487	5.3**
S × T	12	345.4	34.3***	12	123.1	23.1***	12	1277.201	20.5***
SP × S × T	12	197.5	19.6***	12	42.9	8.0***	12	204.869	3.2**

*^∗∗∗^Factors are significant at P < 0.001 level. ^∗∗^Factors are significant at P < 0.01 level. ^∗^Factors are significant at P < 0.05.*

Germination rate was also affected by ryegrass species, temperature, salinity and their interactions (*P* < 0.001, **Table 1**). For both species, similar trends were found between germination rate and percentage. However, the lowest temperature (10–20°C) also had the lowest germination rate in both distilled water and salinity stress treatments (**Figures 1C,D**). Linear regression analysis was used to determine the relationship between germination rate and salinity at different temperatures of ryegrass. There was a strong negative relationship between the germination rate and salinity with coefficient of determination (*R*^2^) ranging from 0.89 to 0.97 under different temperatures (**Figure 3**).

### Interactive Effects of Temperature and Alkalinity on Seed Germination of Ryegrass

**FIGURE 2:**
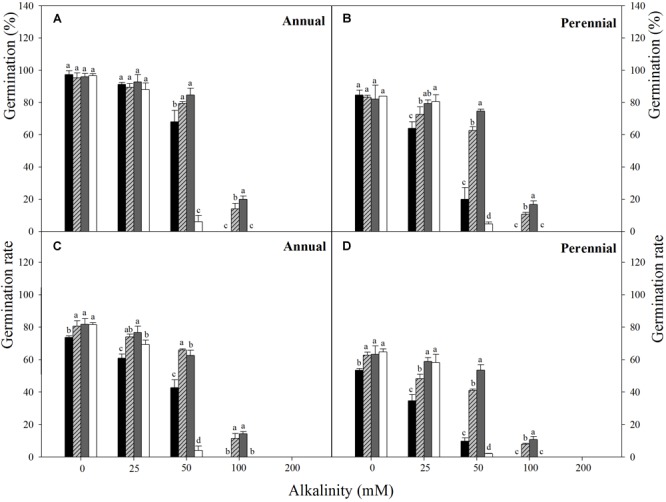
Final germination percentage **(A,B)** and germination rate **(C,D)** of Annual ryegrass **(A,C)** and perennial ryegrass **(B,D)** seeds in alkalinity stress at temperature regimes of 10–20 (■), 15–25 (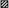), 20–30 (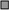), 25–35°C (□). Alkali stress: Na_2_CO_3_. Bars represent mean ± SE (*n* = 3). Different letters indicate significant difference between treatments (Tukey’s test *P* < 0.05).

Three-way ANOVA results showed that seed germination percentage and rate of ryegrass were both affected by ryegrass species, temperature, alkalinity and their interactions (*P* < 0.001, **Table 2**). Seed germination percentage of the two species both decreased with increasing alkalinity at all temperatures, and the changes were much greater than those under salt stress, especially at higher alkali concentrations (100 and 200 mM). For annual ryegrass, no significant difference in seed germination percentage was observed among the four alternating temperatures when the alkalinity was below 50 mM (*P* > 0.05). The optimal temperature for germination was 20–30°C when alkalinity was above 25 mM. When the alkali concentration reached 50 mM, germination percentage at 25–35°C sharply dropped to 6%, but germination percentage at 10–20°C was still 62.6%. In addition, no seed germinated at either 10–20°C or 25–35°C when the alkali concentration was 100 mM, and no seed germinated at all temperatures under the highest alkalinity (200 mM) (**Figure 2A**). For perennial ryegrass, the optimal temperature for germination was 20–30°C under alkali stress. Similar to the annual ryegrass, germination percentages also markedly decreased at 10–20 and 25–35°C when the alkalinity reached 50 mM, and the value was much lower under 25–35°C (**Figure 2B**).

**Table 2:** Three-way ANOVA for the effects of ryegrass species (SP), temperature (T), alkalinity (A) and their interactions on seed germination of ryegrass.

	Germination percentage			Germination rate			Germination recovery		
Independent variable	df	Mean-square	*F*-ratio	df	Mean-square	*F*-ratio	df	Mean-square	*F*-ratio
SP	1	2960.1	311.6***	1	3976.1	714.944***	1	1607.2	30.0***
A	4	41065.1	4322.6***	4	23776.1	4275.154***	4	2433.6	45.5***
T	3	2083.8	219.3***	3	1717.4	308.803***	3	786.1	14.7***
SP × A	4	444.7	46.8***	4	573.4	103.111***	4	886.4	16.6***
SP × T	3	235.4	24.8***	3	152.1	27.344***	3	269.7	5.0**
A × T	12	1303.5	137.2***	12	761.9	136.991***	12	323.9	6.1***
SP × A × T	12	124.1	13.1***	12	59.2	10.648***	12	174.7	3.3**

*^∗∗∗^Factors are significant at P < 0.001 level; ^∗∗^factors are significant at P < 0.01 level.*

**FIGURE 3:**
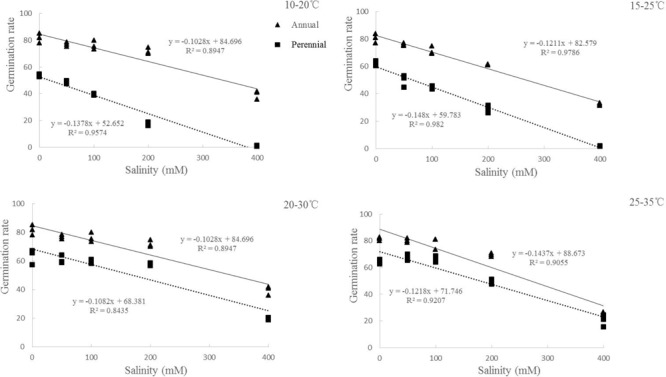
Regression analysis between germination rate and salinity of ryegrass seeds under different temperatures (10–20, 15–25, 20–30, and 25–35°C). (All factors are significant at *P* < 0.05 level).

**FIGURE 4:**
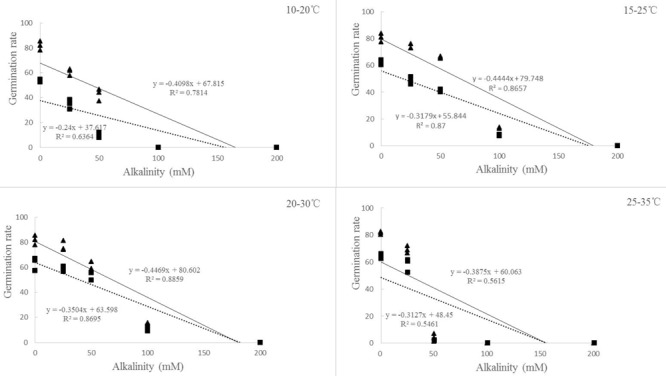
Regression analysis between germination rate and alkalinity of ryegrass seeds under different temperatures (10–20, 15–25, 20–30, and 25–35°C). (All factors are significant at *P* < 0.05 level).

For both of the ryegrass species, similar trend was found between germination rate and percentage. However, the changes of germination rate under alkali stress were different from those under salt stress (**Figures 2C,D**). When the alkali concentration was lower (25 mM), the germination rate at 25–35°C was much higher than that at 10–20°C, but reached the lowest once the alkalinity reached 50 mM. There also appeared to be a negative relationship between germination rate and alkalinity at different temperatures (**Figure 4**).

### Interactive Effects of Temperature and Salinity–Alkalinity on Recovery of Ryegrass

Three-way ANOVA results showed that the percentage of seed germination recovery in ryegrass was affected by ryegrass species, temperature, salinity–alkalinity and their interactions (*P*-value at least below 0.05, **Tables 1, 2**).

For annual ryegrass, the recovery percentage was increased with increasing salinity at 10–20, 15–25, and 25–35°C. When the salinity concentrations were at the high levels (200 and 400 mM), recovery percentages were highest at 10–20°C, which were 50 and 67.8%, respectively. However, seed recovery percentage was very low at 25–35°C, and no seed could germinate again after being transferred to the distilled water when salinity was above 100 mM. Under alkalinity stress, the recovery percentage decreased with increasing alkalinity, and the highest value was found when the seeds germinated at 10–20°C. When the alkalinity reached 200 mM, no seed could recover at any temperature. In addition, the recovery percentages were all 0 under each alkalinity at 25–35°C (**Table 3**).

**Table 3:** Germination recovery percentage (mean ± SE) of ryegrass seeds after transferred to distilled water from salinity stress and alkalinity stress at 10–20, 15–25, 20–30, and 25–35°C.

Species	Stress type	Concentration (mM)	10–20°C	15–25°C	20–30°C	25–35°C
		50 (S1)	8.3 ± 1.3Aa	8.3 ± 1.3Aa	8.3 ± 1.3Aa	19.4 ± 2.2Ab±
	Salinity	100 (S2)	15 ± 2.6Ab	23.3 ± 1.7Bb	44.4 ± 6.0Cb	21.7 ± 3.7Bb±
		200 (S3)	50 ± 4.1Dc	22.4 ± 2.4Bb	41.9 ± 1.0Cb	0Aa
Annual		400 (S4)	67.8 ± 3.6Cd	72.5 ± 2.3Cc	49.5 ± 3.7Bb	0Aa
		25 (A1)	53.3 ± 3.3Bc	44.4 ± 5.6Bc	46.7 ± 5.0Bc	0Aa
	Alkalinity	50 (A2)	19.9 ± 3.1Bb	22.4 ± 2.4Bb	25.9 ± 3.9Bb	0Aa
		100 (A3)	0Aa	2.3 ± 0.1Ba	0Aa	0Aa
		200 (A4)	0Aa	0Aa	0Aa	0Aa

		50 (S1)	13.7 ± 3.3Aa	6.1 ± 1.1Aa	4.8 ± 1.8Aa	0Aa
	Salinity	100 (S2)	26.6 ± 5.5Bb	12.0 ± 2.5Aa	35.9 ± 4.4Bc	0Aa
		200 (S3)	62.9 ± 1.5Cc	30.9 ± 2.5Bb	23.9 ± 3.9Bb	0Aa
Perennial		400 (S4)	63.3 ± 3.5Dc	42.9 ± 3.1Cc	28.8 ± 1.7Bb	8.0 ± 0.3Ab ±
		25 (A1)	17.2 ± 3.3Cb	4.4 ± 0.2ABb	9.8 ± 0.7BCb	0Aa
	Alkalinity	50 (A2)	31.8 ± 2.0Cc	5.4 ± 0.2Bb	0Aa	0Aa
		100 (A3)	0Aa	0Aa	0Aa	0Aa
		200 (A4)	0Aa	0Aa	0Aa	0Aa

*The small letters denote the significant differences among the different stress concentration in the same temperatures; the capital letters denote the significant differences among the different temperatures at same stress concentration. The differences in each parameter were detected by one-way ANOVA at P < 0.05 level. Bars represent mean ± SE (n = 3)*.

Similar results of recovery percentages at different temperatures were also found in perennial ryegrass, and were much higher at 10–20°C but much lower at 25–35°C. In addition, germination recovery percentages were much lower under alkalinity stress. When the alkalinity was only 50 mM, few seeds germinated again. Once the alkalinity was above 50 mM, no seed could germinate again at any temperature (**Table 3**).

## Discussion

Seed germination responses to the environmental changes such as diurnal temperature fluctuations and soil salinity–alkalinity are quite important to determine the colonization capacity of plant species (Gorai and Neffati, 2007), especially in the Northeast of China, where the rainfall is irregular and the potential evapotranspiration always exceeds precipitation. Temperature can profoundly influence either the water absorption speed or the physiological-biochemical processes of cell division within the seed, affecting the seed germination percentage and germination rate (Kermode, 1990).

In this study, the results showed that annual ryegrass and perennial ryegrass both had high germination percentage in distilled water, and had no significant differences among the four temperatures, indicating that temperature is not a limiting factor in non-salinity/alkalinity habitats if soil moisture is enough for the seeds, and ryegrass can adapt to a wide range of temperatures during seed germination stage. In addition, we also found that 20–30°C was the optimal temperature for seed germination of annual ryegrass, and 20–30°C or 25–35°C for the perennial ryegrass, indicating that a relatively higher temperature is beneficial to seed germination of this grass species. Similar results were also found in the research of *Kochia scoparia* (Khan et al., 2001), *Aster tripolium, Triglochin maritimum* (Al-Hawija et al., 2012), and *Leymus chinensis* (Lin et al., 2017). These plants all prefer higher temperatures during the seed germination stage.

Successful establishment of plants largely depends on successful germination, particularly under salt-alkaline condition (Li et al., 2010). It’s generally accepted that osmotic and the ionic effects are the dominating factors that inhibit seed germination behavior under salt stress (Debez et al., 2004). In addition, temperature and salinity have been reported to interact in affecting seed germination of most plant species, and it also has significant ecological implications in terms of the time of germination under field conditions (Ungar, 1995; Khan and Gul, 1998). Our results showed that the optimal temperature (20–30°C) can alleviate the inhibiting effect of salt stress on seed germination of annual ryegrass, especially under the highest salinity (400 mM). Similar trends were also found in perennial ryegrass, where higher temperature regimes (20–30 and 25–35°C) mitigated the detrimental effect of salt stress. The lower temperature aggravated the negative impact under high salinity possible due to that the enzymatic activity that can induce seed germination is relatively low. The low temperature also intensified the osmotic stress and decreased water absorption ability of the seed to some extent. In addition, ryegrass seed germination rate was increased with increasing temperatures under most salinity concentrations. Fast germination at 20–30 and 25–35°C might enhance rapid seedling establishment, which can minimize competition on seedling stage under suitable thermoperiod (Zia and Khan, 2004).

Alkaline stress also significantly inhibited seed germination and germination rate at these four temperature regimes. In the present study, we ensured the same Na^+^ content between the salinity and alkalinity at each stress concentration for the two ryegrass, when the Na^+^ concentration was low (50 mM), no significantly change was found in germination percentage between salt stress and alkali stress at all temperatures, indicating that high pH is not a inhibiting factor for seed germination at low Na^+^ condition. However, with the increasing alkalinity, the inhibiting action of alkaline stress was much greater than that of saline stress at the same Na^+^ concentration. This phenomenon indicates that the interactive effect of Na^+^ and high pH is more harmful to ryegrass seeds than that with only Na^+^ when alkalinity is high, which is consistent with previous reports (Shi and Yin, 1993; Lin et al., 2011; Lin J.X. et al., 2014). However, these results differ from previous studies, where the researchers investigated the comparative effects of salt stress and alkali stress (high pH) on the seedlings of some plant species. They all found that due to the high pH, low concentration alkali stress also strongly inhibited seedling growth, and the detrimental effect was much more markedly than salt stress (Yang et al., 2008; Guo et al., 2009; Zhang and Mu, 2009). Thus, the results also indicate that seed germination and seedling stages have different physiological responses to the salt and alkali stresses. The specific molecule mechanisms need further research.

Additionally, in contrast to temperature-salinity interactions, when the alkalinity concentration reached 50 mM (Na^+^ concentration: 100 mM), germination percentages of the two ryegrass sharply decreased at 25–35°C, indicating that the negative impacts of high pH on germination stage is seriously aggravated at high temperature, and ryegrass seeds are injured more under alkalinity stress at higher temperatures. This result was mainly because of the irreversible damage caused by the toxicity of Na^+^ at higher temperatures (Khan and Ungar, 1996). However, this inhibition is also a physiological adaptive strategy of seeds to resist to the high temperature-alkalinity stresses. Seed germination is likely to occur when environments favor the seedlings survived, and high temperatures can prevent seed germination and avoids seedling mortality under extreme conditions. Compared with annual ryegrass, germination percentage and rate were both lower in perennial ryegrass, and besides the highest temperature (25–35°C), the lowest temperature (10–20°C) also lead to a markedly decrease in the seed germination percentage at 50 mM alkalinity stress, indicating that alkali-tolerance of perennial ryegrass is weaker than that of annual ryegrass, since both higher or lower temperatures reduced the alkali-tolerance of this species. Annual and perennial ryegrasses are two closely related species, but annual ryegrass must complete its life cycle under adverse conditions in only a year. Thus, annual ryegrass is more tolerant to unbefitting temperature and soil conditions, and occupies its own ecological niche in limited time compared with perennial ryegrass.

The ability of plant seeds to survive in salinity conditions and germinate recovery when the soil salinity is reduced provides them with opportunities for the following seedling establishment in unpredictable salt-alkaline environment (Khan and Ungar, 1997; Song et al., 2006; Qu et al., 2008). Ryegrass seeds also showed some recovery ability though germination recovery percentage was significantly affected by the temperature, salinity, alkalinity, and their interactions. In our study, we clearly found that for both ryegrass species, recovery percentages reached highest at the highest salinity (400 mM), but the lower concentrations under alkalinity stress (25 and 50 mM). The highest germination percentage in salt stress indicates that short duration salinity does not affect seed viability, and ryegrass can keep viable and germinate again when salinity concentration was alleviated. The difference in recovery behavior between salinity and alkalinity stress is possibly due to the high pH of alkaline solutions. The lower recovery percentage at higher alkalinity compared to salinity also confirms this viewpoint. Thus, different inhibition mechanisms on seed germination of this species may exist between the two stresses, which deserve further research.

Results from our present study also indicate that germination recovery percentages of ryegrass are much higher at 10–20°C under most salinity–alkalinity concentrations. However, above germination test results have demonstrated that higher temperatures (20–30 and 25–35°C) are more favorable for seed germination of ryegrass. Similar results were found in *Diplotaxis* seeds by Tlig et al. (2008). They found the optimal temperature for seed germination of this species is a constant temperature of 15°C, but 5 and 10°C for recovery germination. However, this phenomenon is in contradiction to the observations reported in seeds recovery of *K. scoparia* (Khan et al., 2001), *Salsola vermiculata* (Guma et al., 2010), and *Leymus chinensis* (Lin et al., 2011). The optimum temperature for seed germination of these species is also suitable for germination recovery. The reason of this difference is likely due to that seeds incubated under high temperatures with salinity–alkalinity concentrations seemed to be subjected to more irreversible damage, which decreases the germination recovery capacity of the seeds, and the reason is worthy of further study and exploitation. Thus, for ryegrass, higher temperature (20–30°C) is beneficial to germination under salt and alkali stresses environments, while the lower temperature (10–20°C) is beneficial to germination recovery when salt-alkaline condition is relieved.

## Conclusion

This paper first reported germination responses of both annual ryegrass and perennial ryegrass seeds to the interactive effects of temperature and salt-alkali stress, and also provided a new insight to cognize abiotic stress tolerance in two closely related species during germination stage. Our results clearly showed that salt stress and alkali stress are actually two different stress types, and the salt-alkali tolerance of ryegrass seeds is greatly affected by the interactions of temperature and salinity–alkalinity.

## Author Contributions

JL wrote the manuscript. XH did the experiments. XP made the figures. XY revised the manuscript. BD revised the language mistakes and gave me some good advice on how to revise the paper.

## Conflict of Interest Statement

The authors declare that the research was conducted in the absence of any commercial or financial relationships that could be construed as a potential conflict of interest.
